# Dairy consumption and risk of esophagus cancer in the prostate, lung, colorectal, and ovarian cohort

**DOI:** 10.3389/fnut.2022.1015062

**Published:** 2022-12-08

**Authors:** Tingting Wang, Yi Zhu, Yuanzhu Zheng, Yang Cao, Qin Xu, Xiangan Wang, Wenli Hu, Yun Zhang

**Affiliations:** ^1^Department of Nutrition, The First Affiliated Hospital, College of Medicine, Zhejiang University, Hangzhou, Zhejiang, China; ^2^Department of Communicable Diseases Control and Prevention, Hangzhou Center for Disease Control and Prevention, Hangzhou, Zhejiang, China; ^3^Department of Hepatobiliary and Pancreatic Surgery, The First Affiliated Hospital, College of Medicine, Zhejiang University, Hangzhou, Zhejiang, China

**Keywords:** dairy product, esophagus cancer, PLCO, cohort, risk

## Abstract

**Background:**

Epidemiological studies provide limited information on the relationship between dairy consumption and the incidence of esophagus cancer (EC). We examined whether eating dairy foods is associated with a lower risk of EC in an American population.

**Methods:**

In our study, we analyzed data from the Prostate, Lung, Colorectal, and Ovarian (PLCO) cancer screening trial, which included 101,723 subjects. Dairy product consumption was assessed using a dietary history questionnaire. We used Cox regression and restricted cubic splines to assess whether dairy consumption is associated with EC incidence.

**Results:**

A total of 154 EC cases were identified after a median follow-up of 12.2 years. After adjusting for confounders, we discovered no statistically significant correlation between total dairy product consumption and EC risk (HR with 95% CI for ≥1.79 servings/day vs. ≤0.6 servings/day: 0.83, 0.50–1.38; *p* for trend = 0.465). Additionally, no associations were found between EC risk and other dairy foods such as milk, yogurt, and cheese.

**Conclusion:**

We concluded that the findings of the PLCO cohort do not suggest dairy consumption reduces the risk of EC.

## Introduction

Esophagus cancer (EC) is the seventh most prevalent and sixth most deadly cancer type. In 2018, the estimated number of cases worldwide was 572,000 (1). The common histological types of EC are esophageal squamous cell carcinoma (ESCC) and esophageal adenocarcinoma (EAC). ESCC, which develops in the upper part of the esophagus, is prevalent worldwide. EAC, which occurs at the esophageal-stomach junction, is more common in developed countries such as the United States and England (2). EC has disproportionately high mortality rates because by the time it is identified, the cancer has advanced or metastasized. In the American population, only 18% of EC patients have cancer at the primary site, while 40% of cases have distant metastasis, which is associated with a terrible prognosis (3). Consequently, exploration of the etiological factors and prevention methods is crucial in EC. Risk factors include obesity in EAC (4, 5), alcohol drinking, and maté consumption (a kind of piping hot tea drank in South America) in ESCC (6, 7). There is limited evidence that lack of physical activity, low vegetable and fruit consumption, and high processed meat consumption may increase the risk of EC (2).

Milk products are consumed all over the world in a variety of forms, including milk, yogurt, cheese, and butter. Milk has been extensively researched in relation to the causes of colon, prostate, and breast cancers (8–10). However, there is limited evidence on the correlation between dairy foods and EC risk. Previous epidemiological researches have demonstrated a negative correlation between dairy foods and EC risk (11, 12). Li et al. pointed out that yogurt intake may offer a protective effect for EC (13). However, two cohort studies indicated that there was no relationship between different dairy products and EC risk (14, 15). In this study, we explored whether consuming dairy foods is linked to a lower risk of EC in an American population.

## Materials and methods

### Study population

The PLCO trial, which involves a sizable population for cancer screening, provided the data for our study. The trial evaluated whether screening exams could lower the mortality risk for prostate, lung, colorectal, and ovarian cancers (16). Between 1993 and 2001, the study involved 154,887 individuals recruited from ten centers in the US. The inclusion criteria were the following: (a) individuals aged 55–74 years; (b) who completed the basic questionnaire, (c) and also provided informed consent. And the exclusion criteria were those who (a) had a history of prostate, lung, colorectal, or ovarian cancers; (b) were joining other trials, (c) were undergoing treatment for cancer, (d) received a recent screening examination for colorectal or prostate cancer.

In our study, participants were further excluded if they (a) failed to finish the diet history questionnaire (DHQ), (b) were given a cancer diagnosis before completing the DHQ, (c) did not finish the baseline questionnaire, or (d) were diagnosed with EC or died from EC between the baseline questionnaire completion and the DHQ completion. The Institutional Review Board at the National Cancer Institute approved the research after receiving signed informed permission from each participant.

### Data collection

A self-reported baseline questionnaire was used to gather baseline data, which included demographics (e.g., gender, race, and education), cigarette smoking, and marital status. The DHQ was used to gather the remaining baseline data, which included the participant’s age at the completion of the questionnaire, alcohol consumption, and calorie intake. Through the DHQ, participants reported consuming 124 different foods, including dairy products, throughout the previous year (17). Participants needed to answer “How frequently did you drink milk as a beverage?”. There were ten predefined answers: never, once per month or less, twice or three times per month, once or twice per week, three or four times per week, five or six times per week, once per day, twice or three times per day, four or five times per day, and six or more times per day. Milk consumers further needed to answer the question of how much to drink each time. The predefined answers were less than one cup, one to one and a half cups, and more than one and a half cups. Participants also needed to provide the frequency and intake of yogurt and cheese. The daily dairy consumption was calculated by DietCalc software according to the pyramid servings of the US Department of Agriculture, which were based on national dietary data from 1994 to 1996 (18).

### Ascertainment of esophagus cancer

Subjects were followed up through an annual questionnaire to screen for cancer cases. They needed to provide information on method of diagnosis such as histology, cytology, radiology, or others. Participants also needed to answer the question about the ICD-O-2 cancer classification of primary cancer. End points for cancer incidence were confirmed invasive tumors, cancers *in situ*, and borderline malignancies. The medical records were reviewed in order to assess the EC cases. In this study, EC was diagnosed using codes C15.0–C15.9 based on the International Classification of Disease for Oncology, Second Edition [ICD-O-2].

### Statistical analysis

We used the packages R,^1^ Stata MP software version 17.0, and SPSS software version 24.0 for our statistical study. According to participants’ dairy intake, baseline characteristics were presented by quartile (Q), with cutoff values chosen based on the distribution of the total cohort. To assess differences in variables among four groups, we used the ANOVA and Chi-square tests, respectively. Under two-tailed analysis, we considered *p* < 0.05 to be statistically significant.

We used Cox proportional hazards models to assess whether dairy consumption could reduce the risk of EC. In both non-adjusted and multivariate-adjusted models, the hazard ratios (HR) and 95% confidence intervals (95% CI) were presented. We used Schoenfeld residuals to examine the proportional hazards assumption before modeling Cox regression. We constructed sex stratification for Cox proportional hazards models because it violated the proportional hazards assumption in multivariable analyses. Confounders were assessed by either adding variables to a basic Cox regression model or by removing each covariate from the whole model, respectively. Then, we compared the regression coefficients and chose the factors that modified the original regression coefficients by more than 10% (19). We obtained confounders from the literature and clinical judgment. Specifically, model 1 was stratified by gender and adjusted for age, while model 2 was stratified by gender and adjusted for age, alcohol drinking status, cigarette smoking status, body mass index (BMI), occupation, and total calorie intake. The missing data was less than 6%; therefore no imputation was performed. In order to provide a more comprehensive elaboration, we applied multiple linear (Cox regression) and non-linear (restricted cubic spline, RCS) statistical methods to evaluate the intricate exposure-response relationship between dairy consumption and EC risk. Dairy consumption was treated with four knots at the 5th, 35th, 65th, and 95th percentiles in the restricted cubic spline model (20). To minimize potential effects on the findings, we excluded extreme values of dairy consumption (less than 1% or more than 99%) before the dose-response analysis. We investigated the null hypothesis that the regression coefficients of the second and third splines were equal to zero in order to obtain *p* for non-linearity (21).

We evaluated the stability of the findings using sensitivity analyses. We repeated Cox regression model 2 with the following modifications: (a) excluding male participants with extreme calorie intakes of less than 800 kcal per day or more than 4,000 kcal per day and female participants with calorie intakes of less than 500 kcal per day or more than 3,500 kcal per day (22); and (b) excluding patients diagnosed with EC within the first two years.

## Results

We diagnosed a total of 154 EC cases from 101,723 participants after a median follow-up of 12.2 years. The number of EC cases in each grade was as follows: 39 cases were undetermined or unstated; 5 cases were undifferentiated in Grade IV; 61 cases were poorly differentiated in Grade III; 43 cases were moderately differentiated in Grade II; and 6 cases were highly differentiated in Grade I. Among them, 3 cases were *in situ* cancers, and 151 cases were malignant, primary site cancers. Table 1 shows the characteristics of subjects according to quartiles of dairy product consumption. The subjects in the highest quartile (1.79 servings/day) were more likely to be older, male, white, non-smokers, have a higher level of education (college graduate and postgraduate), and a high total calorie intake compared with subjects in the other three quartiles.

**TABLE 1:** Baseline characteristics of study population according to total dairy consumption in 101,723 participants.

		Quartile (Q) of dairy consumption, servings/day				
Characteristics^a^	Overall	Q1(≤0.6)	Q2(0.61–1.06)	Q3(1.07–1.78)	Q4(≥1.79)	*p*
Number of participants	101723	25675	25212	25637	25199	
Age at the diet history questionnaire completion (years)	65.53 ± 5.73	65.53 ± 5.69	65.49 ± 5.72	65.45 ± 5.76	65.64 ± 5.75	0.002
BMI (kg/m^2^)	27.23 ± 4.82	26.99 ± 4.88	27.28 ± 4.83	27.31 ± 4.81	27.35 ± 4.74	<0.001
Energy intake (kcal/day)	1738.64 ± 736.42	1328.98 ± 563.83	1605.98 ± 600.34	1846.26 ± 678.21	2179.28 ± 801.42	<0.001
**Sex**						
Male	49477 (48.64)	10836 (42.20)	11746 (46.59)	12927 (50.42)	13968 (55.43)	<0.001
Female	52246 (51.36)	14839 (57.80)	13466 (53.41)	12710 (49.58)	11231 (44.57)	
**Smoking status**						
Never	48548 (47.73)	11835 (46.10)	11967 (47.47)	12210 (47.63)	12536 (49.75)	<0.001
Current	9399 (9.24)	2731 (10.64)	2084 (8.27)	2224 (8.67)	2360 (9.37)	
Former	43763 (43.02)	11107 (43.26)	11158 (44.26)	11200 (43.69)	10298 (40.87)	
Unknown	13 (0.01)	2 (0.01)	3 (0.01)	3 (0.01)	5 (0.02)	
**Alcohol drinking status**						
Never	10113 (9.94)	2645 (10.30)	2260 (8.96)	2387 (9.31)	2821 (11.19)	<0.001
Current	73973 (72.72)	18221 (70.97)	18814 (74.62)	19017 (74.18)	17921 (71.12)	
Former	14756 (14.51)	3928 (15.30)	3450 (13.68)	3549 (13.84)	3829 (15.20)	
Unknown	2881 (2.83)	881 (3.43)	688 (2.73)	684 (2.67)	628 (2.49)	
**Education**						
College below	64733 (63.64)	17458 (68.00)	16100 (63.86)	15817 (61.70)	15358 (60.95)	<0.001
College graduate	17845 (17.54)	3982 (15.51)	4366 (17.32)	4759 (18.56)	4738 (18.80)	
Postgraduate	18948 (18.63)	4169 (16.24)	4703 (18.65)	5013 (19.55)	5063 (20.09)	
Unknown	197 (0.19)	66 (0.26)	43 (0.17)	48 (0.19)	40 (0.16)	
**Race**						
White, Non-hispanic	92503 (90.94)	20905 (81.42)	23139 (91.78)	24237 (94.54)	24222 (96.12)	<0.001
Black, Non-hispanic	3352 (3.29)	1697 (6.61)	780 (3.09)	533 (2.08)	342 (1.36)	
Hispanic	1495 (1.47)	496 (1.93)	406 (1.61)	313 (1.22)	280 (1.11)	
Other^b^	4336 (4.26)	2562 (9.98)	879 (3.49)	545 (2.13)	350 (1.39)	
Unknown	37 (0.04)	15 (0.06)	8 (0.03)	9 (0.04)	5 (0.02)	
**Occupation**						
Not working	12723 (12.51)	3414 (13.30)	3228 (12.80)	3094 (12.07)	2987 (11.85)	<0.001
Working	40714 (40.02)	10147 (39.52)	9916 (39.33)	10486 (40.90)	10165 (40.34)	
Retired	43704 (42.96)	10916 (42.52)	10998 (43.62)	10933 (42.65)	10857 (43.09)	
Other^c^	1058 (4.05)	1082 (4.21)	996 (3.83)	1007 (3.93)	1068 (4.24)	
Unknown	459 (0.45)	116 (0.45)	104 (0.41)	117 (0.46)	122(0.48)	
**Randomization arm**						
Intervention	51805 (50.93)	12853 (50.06)	12902 (51.17)	13146 (51.28)	12904 (51.21)	0.016
Control	49918 (49.07)	12822 (49.94)	12310 (48.83)	12491 (48.72)	12295 (48.79)	
**Marital status**						
Married	98321(96.66)	24802 (96.60)	24398 (96.77)	24776 (96.64)	24345 (96.61)	0.523
Not married	3216 (3.16)	821 (3.20)	771 (3.06)	807 (3.15)	817 (3.24)	
Unknown	186 (0.18)	52 (0.20)	43 (0.17)	54 (0.21)	37 (0.15)	
**Family history of esophagus cancer**						
Yes	813 (0.80)	233 (0.91)	195 (0.77)	195 (0.76)	190 (0.75)	0.210
No	97478 (95.83)	24568 (95.69)	24190 (95.95)	24601 (95.96)	24119 (95.71)	
Possibly	2658 (2.61)	691 (2.69)	648 (2.57)	645 (2.52)	674 (2.67)	
Unknown	774 (0.76)	183 (0.71)	179 (0.71)	196 (0.76)	216 (0.86)	

BMI, body mass index.

^a^Data are presented as mean ± standard deviation or counts (percentage).

^b^“Other” refers to Asian, Pacific Islander and American Indian.

^c^“Other” refers to extended sick leave, disabled.

The findings of dairy consumption and EC incidence using univariable and multivariable Cox regression are presented in Table 2. The HR with 95% CI of EC in the fourth quartiles of total dairy consumption (1.79 servings/day) compared to those in the first quartiles (0.6 servings/day) in the multivariate-adjusted model was 0.83, 0.50–1.38 (*p* for trend = 0.465). We also found no statistically significant associations between EC risk and different dairy products such as milk, yogurt, and cheese.

**TABLE 2:** Association between dairy consumption and esophagus cancer risk in the PLCO trial.

Variables (servings/day)	Cases (n)	Person- years	Incidence rate/10,000 person-years	Cox proportional hazards regression (HR, 95% CI)		
				Unadjusted model	Adjusted model 1^a^	Adjusted model 2^b^
**Total dairy products**						
Q1(≤0.6)	39	226237.11	1.72	Reference group	Reference group	Reference group
Q2(0.61–1.06)	37	223812.13	1.65	0.96 (0.61–1.50), *p* = 0.852	0.89 (0.57–1.40), *p* = 0.608	0.96 (0.61–1.53), *p* = 0.866
Q3(1.07–1.78)	40	227364.74	1.76	1.02 (0.66–1.59), *p* = 0.929	0.90 (0.58–1.40), *p* = 0.628	0.92 (0.57–1.47), *p* = 0.716
Q4(≥ 1.79)	38	223488.36	1.70	0.99 (0.63–1.54), *p* = 0.952	0.81 (0.52–1.27), *p* = 0.355	0.83 (0.50–1.38), *p* = 0.477
				*p* for trend = 0.976	*p* for trend = 0.387	*p* for trend = 0.465
**Milk**						
Q1(≤ 0.3)	38	228373.45	1.66	Reference group	Reference group	Reference group
Q2(0.31–0.66)	41	223526.50	1.83	1.10 (0.71–1.71), *p* = 0.672	0.95 (0.61–1.48), *p* = 0.830	0.96 (0.61–1.51), *p* = 0.858
Q3(0.67–1.3)	37	224619.60	1.65	0.99 (0.63-1.55), *p* = 0.960	0.79 (0.50–1.25), *p* = 0.314	0.84 (0.53–1.34), *p* = 0.461
Q4(≥ 1.31)	38	224382.79	1.69	1.02 (0.65–1.59), *p* = 0943	0.77 (0.49–1.21), *p* = 0.258	0.76 (0.47–1.24), *p* = 0.278
				*p* for trend = 0.935	*p* for trend = 0.184	*p* for trend = 0.227
**Yogurt^#^**						
Q1(0)	98	401141.62	2.44	Reference group	Reference group	Reference group
Q2(0.01–0.02)	24	194938.22	1.23	0.50 (0.32–0.79), *p* = 0.003	0.68 (0.44–1.07), *p* = 0.098	0.68 (0.43–1.08), *p* = 0.103
Q3(0.03–0.06)	19	141768.02	1.34	0.55 (0.34–0.9), *p* = 0.016	0.86 (0.52–1.41), *p* = 0.536	0.94 (0.57–1.55), *p* = 0.808
Q4(≥ 0.07)	13	163054.48	0.80	0.33 (0.18–0.58), *p*<0.001	0.57 (0.32–1.03), *p* = 0.062	0.62 (0.35–1.13), *p* = 0.118
				*p* for trend <0.001	*p* for trend = 0.051	*p* for trend = 0.126
**Cheese**						
Q1(≤0.1)	46	239510.73	1.92	Reference group	Reference group	Reference group
Q2(0.11–0.2)	35	219272.34	1.60	0.83 (0.54–1.29), *p* = 0.411	0.83 (0.53–1.29), *p* = 0.408	0.80 (0.50–1.26), *p* = 0.329
Q3(0.21–0.37)	35	218078.51	1.60	0.84 (0.54–1.30), *p* = 0.426	0.81 (0.52–1.26), *p* = 0.354	0.76 (0.48–1.22), *p* = 0.260
Q4(≥ 0.38)	38	224040.76	1.70	0.88 (0.58–1.36), *p* = 0.576	0.79 (0.51–1.23), *p* = 0.299	0.75 (0.46–1.22), *p* = 0.246
				*p* for trend = 0.578	*p* for trend = 0.302	*p* for trend = 0.241

PLCO, prostate, lung, colorectal, and ovarian cancer; HR, hazard ratio; 95% CI, 95% confidence interval; Q, quartile; BMI, body mass index.

^a^Stratified by sex (male, female) due to proportional hazard assumption violation and adjusted for age (years).

^b^Stratified by sex (male, female) due to proportional hazard assumption violation and adjusted for age (years), alcohol drinking status (never, current, former), smoking status (never, current, former), occupation (not working, working, retired, others), body mass index (<25 kg/m^2^, ≥25kg/m^2^), total calorie intake (kcal/day).

^#^The first category was non-consumers, with the remaining consumers classified as tertiles of distribution.

**FIGURE 1:**
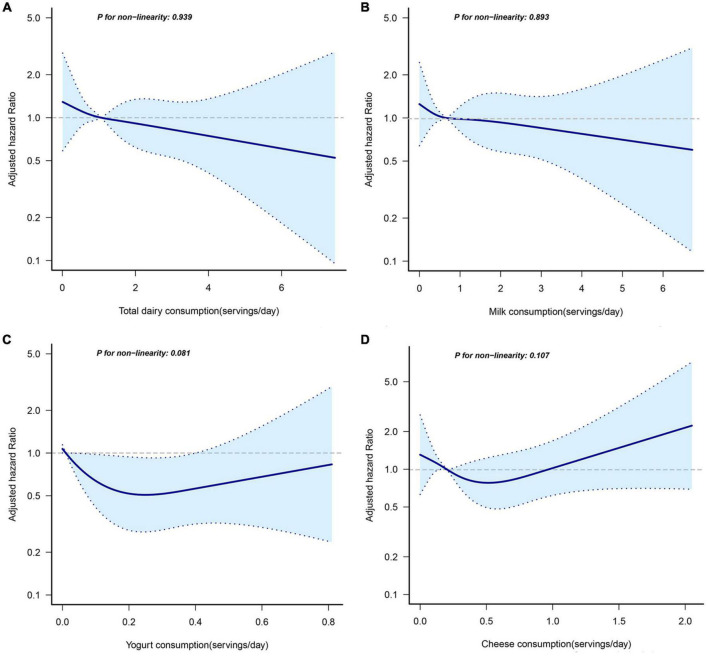
Non-linear dose-response analysis on total dairy products **(A)**, milk **(B)**, yogurt **(C)**, and cheese **(D)** consumption and incidence of EC. Hazard ratios were calculated by restricted cubic spline regression with four knots (i.e., the 5th, 35th, 65th, and 95th percentiles) after adjusting for age (years), alcohol drinking status (never, current, former), smoking status (never, current, former), occupation (not working, working, retired, others), body mass index (<25 kg/m^2^, ≥25 kg/m^2^), total calorie intake (kcal/day). The solid line represents a non-linear trend, and dashed lines represent 95% confidence intervals.

The RCS regression plots evaluating the non-linear association between dairy consumption and EC risk are shown in Figure 1. However, there was no evidence of a non-linear correlation between consumption of total or individual dairy products and EC risk.

Sensitivity analyses confirmed that our findings were stable. Dairy consumption and EC incidence did not change when we eliminated the 27 cases reported within the first two years. Furthermore, the results remained unchanged after excluding 2889 participants with extreme calorie intake (Supplementary Table 1).

## Discussion

In multivariable adjusted models, we did not find any statistically significant correlations between dairy product consumption and EC risk in this large prospective population. These findings persisted in sensitivity analysis, demonstrating the validity of our conclusion.

Protein, fat, and minerals are abundant in dairy products. Researchers have investigated the role of milk in the etiology of colon, breast, and prostate cancers (23–25); however, there is no information on its association with EC risk in the US. According to a 2016 report from the World Cancer Research Fund and the American Institute for Cancer Research, there is “limited-no conclusion” regarding the link between dairy consumption and EC. To date, it has been unknown whether dairy product consumption could decrease the risk of EC. The meta-analyses has reported that fermented dairy food consumption, including cheese and yogurt, have a protective effect on EC (26); however, there is no association between drinking milk and the incidence of EC (13). Yogurt consumption is inversely associated with the risk of ESCC in a Japanese population (27). A case-control study from Ireland showed no significant associations between dairy product consumption and EAC risk (28). However, another case-control study from a Chinese population found that milk or dairy products were positively associated with an increased the risk of EC (29). These studies were retrospective; prospective studies provided limited and inconsistent evidence. Milk had a protective role against the development of EC in a Shanghai population cohort (30). The results of our study were consistent with the Japanese cohort study, which prospectively showed that milk intake was not significantly correlated to EC risk (15).

The evidence for dairy products either preventing cancer or increasing cancer incidence is inconclusive. Dairy products may be positively or negatively associated with cancer through their constituents or metabolites, including protein, calcium, vitamin D, saturated fatty acids, and butyrate (31–33). Fermented milk products such as yogurt and cheese may be responsible for dairy products’ protective effect on EC risk (26). *Lactobacilli* and *Bifidobacteria*, probiotic microbes found in fermented dairy, have been linked to a variety of healthy benefits, including cancer prevention (34). Both probiotics have been found to have multiple mechanisms against cancer. They have been shown to inhibit pernicious bacterial growth in order to reduce carcinogenic enzymes such as β-glycosidase and azoreductase (35). They have been discovered to exert anti-mutagenicity against mutagenic substances *in vitro* (36). They could also inhibit proliferation or induce apoptosis in cancer cells (37, 38).

Furthermore, dietary components such as protein in milk may have a significant impact on cancer by targeting gut microbiota (39). Recent research has shown a high protein diet increases gut *Bifidobacteria* composition in rats (40). Protein can also be fermented by microbes that colonize the intestinal tract. Short chain fatty acids (SCFAs), which are protein metabolites, can alter gut microbiota and show anticancer activity, thus enhancing host defense and immunity (41, 42). SCFAs have been found to inhibit the activity of the enzyme histone deacetylase, which may increase the number of regulatory T cells and the production of interleukin-10 and transforming growth factor-β, promoting cancer cell apoptosis (43).

Although cheese is a fermented milk product, it has a higher fat content than whole milk. It has been observed that high dietary fat intake, especially high-fat dairy product consumption, is significantly related to a higher incidence of EC (44, 45). Indeed, a high-fat diet may cause changes in the esophageal microbiota, particularly affecting the synthesis of SCFAs and bile acids (46). Since 1970, the proportion of Americans drinking whole milk has decreased, while the consumption of cheese has increased by twofold (47), which indicates high-fat dairy consumption may be positively associated with EC risk.

Dairy consumption may have detrimental effects on cancer risk due to the presence of contaminants, including carcinogenic environmental contaminants, pesticides, and mycotoxins (48). In 1997, Schecter et al. discovered that polychlorinated biphenyls (PCBs) and organochlorine pesticides such as DDT, which is now banned in the United States, significantly contribute to dioxin toxic equivalents (TEQs) in butter and cheese (49). According to a US national report on toxic pollutants in milk, the levels of TEQs in milk and dairy products had decreased in 2003 (50). Moreover, dairy contamination with mycotoxins is a risk factor for EC. The mycotoxins, like aflatoxins B1 and M1, that have been found in milk are produced by the molds in the contaminated cattle feed. Aflatoxin B1 is categorized as a powerful human carcinogen. Despite exposure to very low levels of aflatoxin B1, it may have a negative effect on human health (51). In Turkey, another mycotoxin was reported, indicating EC is caused by consumption of moldy cheese in Eastern Anatolia (52).

Dairy products may have both positive and negative connections with the development of different cancers. However, the majority of negative effects for people can result from excessive or indiscriminate consumption. The Cancer Council and the United States Department of Agriculture have recommended three servings of low-fat or fat-free dairy products daily as an important part of a nutritious diet in our lives.

Our study used a prospective cohort design and long follow-up periods to minimize the selection bias. Additionally, dairy product consumption was assessed using a DHQ, which evaluated different dairy products, including non-fermented and fermented milk products. However, there were some limitations in our study. First, the DHQ may lead to recall bias, affect HR, and confounder estimations (53). Second, dairy consumption was evaluated only once at baseline. It was not possible to assess subsequent changes in diet. Third, there may be residual confounders in observational studies. Although potential confounders were adjusted in the PLCO trial, we cannot exclude the possibility that our results were distorted by unrecognized confounders. The last was the low incidence of EC; thus, the insignificant association may be partly due to the reduced statistical power.

In conclusion, we concluded that the findings of the PLCO cohort do not suggest dairy consumption reduces the risk of EC. In addition, further research on different populations is also required to verify our findings.

## Data availability statement

Publicly available datasets were analyzed in this study. This data can be found here: https://biometry.nci.nih.gov/cdas/plco/.

## Ethics statement

The studies involving human participants were reviewed and approved by the Institutional Review Boards at the National Cancer Institute. The patients/participants provided their written informed consent to participate in this study.

## Author contributions

TW and YZa developed the hypothesis, study design, and concept. TW acquired the original data, drafted the initial manuscript, and other authors made critical comments and revisions. TW, YZh, YZe, YC, QX, XW, and WH were responsible for statistical analyses and figure preparation and edition. YZh acted as guarantors for the integrity of the data and the accuracy of the statistical analysis. All authors interpreted the results together, agreed to be totally responsible for ensuring the accuracy and integrity of the work, and read and approved the final manuscript.

## Footnotes

1
http://www.R-project.org
