# Supplementary material for: Dairy consumption and risk of esophagus cancer in the prostate, lung, colorectal, and ovarian cohort
Source: Front Nutr. 2022 Dec 8;9:1015062. doi: 10.3389/fnut.2022.1015062 (PMC9773090; doi:10.3389/fnut.2022.1015062)
Supplement: Supplementary file 1 [file Table_1.docx]

Supplementary Material

**Supplementary Table 1.** Sensitivity analyses on the association between dairy consumption and EC incidence

| Quartile of dairy products (servings/day) | Hazard ratio (95% confidence interval) ^a^ | | |
| --- | --- | --- | --- |
|  | Primary analysis | Sensitivity analyses | |
|  |  | Excluding cases occurring within the first two years (n=27) | Excluding subjects with extreme calorie intake (n=2889) ^b^ |
| Total dairy products |  |  |  |
| Q1(≤0.6) | Reference group | Reference group | Reference group |
| Q2(0.61–1.06) | 0.96 (0.61–1.53), *p* = 0.866 | 0.97 (0.58–1.61), *p* = 0.904 | 0.95 (0.59–1.51), *p* = 0.816 |
| Q3(1.07–1.78) | 0.92 (0.57–1.47), *p* = 0.716 | 0.88 (0.52–1.48), *p* = 0.623 | 0.90 (0.55–1.46), *p* = 0.665 |
| Q4(≥1.79) | 0.83 (0.50–1.38), *p* = 0.477 | 0.83 (0.48–1.46), *p* = 0.523 | 0.87 (0.52–1.46), *p* = 0.591 |
|  | *p* for trend = 0.465 | *p* for trend = 0.474 | *p* for trend = 0.570 |
| Milk |  |  |  |
| Q1(≤0.3) | Reference group | Reference group | Reference group |
| Q2(0.31–0.66) | 0.96 (0.61–1.51), *p* = 0.858 | 0.81 (0.49–1.34), *p* = 0.407 | 0.98 (0.62–1.56), *p* = 0.943 |
| Q3(0.67–1.3) | 0.84 (0.53–1.34), *p* = 0.461 | 0.80 (0.48–1.32), *p* = 0.382 | 0.86 (0.53–1.39), *p* = 0.860 |
| Q4(≥1.31) | 0.76 (0.47–1.24), *p* = 0.278 | 0.79 (0.47–1.34), *p* = 0.204 | 0.82 (0.50–1.35), *p* = 0.818 |
|  | *p* for trend = 0.227 | *p* for trend = 0.227 | *p* for trend = 0.355 |
| Yogurt |  |  |  |
| Q1(0) | Reference group | Reference group | Reference group |
| Q2(0.01–0.02) | 0.68 (0.43–1.08), *p* = 0.103 | 0.64 (0.38–1.07), *p* = 0.089 | 0.67 (0.42–1.08), *p* = 0.099 |
| Q3(0.03–0.06) | 0.94 (0.57–1.55), *p* = 0.808 | 0.88 (0.51–1.52), *p* = 0.647 | 0.88 (0.53–1.47), *p* = 0.624 |
| Q4(≥0.07) | 0.62 (0.35–1.13), *p* = 0.118 | 0.59 (0.31–1.12), *p* = 0.109 | 0.62 (0.34–1.12), *p* = 0.114 |
|  | *p* for trend = 0.126 | *p* for trend = 0.099 | *p* for trend = 0.100 |
| Cheese |  |  |  |
| Q1(≤0.1) | Reference group | Reference group | Reference group |
| Q2(0.11–0.2) | 0.80 (0.50–1.26), *p* = 0.329 | 0.96 (0.58–1.58), *p* = 0.860 | 0.82 (0.51–1.30), *p* = 0.390 |
| Q3(0.21–0.37) | 0.76 (0.48–1.22), *p* = 0.260 | 0.96 (0.58–1.61), *p* = 0.882 | 0.78 (0.48–1.26), *p* = 0.305 |
| Q4(≥0.38) | 0.75 (0.46–1.22), *p* = 0.246 | 0.83 (0.48–1.46), *p* = 0.527 | 0.75 (0.45–1.24), *p* = 0.257 |
|  | *p* for trend = 0.241 | *p* for trend = 0.564 | *p* for trend = 0.256 |

^a^ Stratified by sex (male, female) due to proportional hazard assumption violation and adjusted for age (years), alcohol drinking status (never, current, former), smoking status (never, current, former), occupation (not working, working, retired, others), body mass index (<25 kg/m^2^, ≥25kg/m^2^), total calorie intake (kcal/day).

^b^ Extreme calorie intake is defined as less than 800 or more than 4000 kcal per day for men and less than 500 or more than 3500 kcal per day for women. The excluded subjects (n = 2889) consist of 6 subjects with EC and 2883 subjects without EC.
